# Discovery of Novel Minor Steviol Glycoside from the *Stevia rebaudiana*: Structural Characterization and Proposed Biosynthetic Pathway of Rebaudioside *D17*

**DOI:** 10.3390/biom16010146

**Published:** 2026-01-14

**Authors:** Xiao Juie Wong, Khairul Nizam Bin Nawi, Yeen Yee Wong, Ismail Ammar Bin Mohamat, Saravanan Ramandach, Mohamad Afzaal Bin Hasim, Avetik Markosyan

**Affiliations:** PureCircle Sdn. Bhd., Lengkuk Teknologi, Bandar Enstek 71760, Negeri Sembilan, Malaysia; alicexiaojuie.wong@ingredion.com (X.J.W.); khairul.nizambinnawi@ingredion.com (K.N.B.N.); wong.yeenyee@ingredion.com (Y.Y.W.); ismail.ammar@ingredion.com (I.A.B.M.); ramandach.saravanan@ingredion.com (S.R.); mohamad.afzaalbinhasim@ingredion.com (M.A.B.H.)

**Keywords:** steviol glycoside, Rebaudioside *D17*, diterpene glycosides, high intensity sweeteners, β-1→4 glycosidic linkage, *Stevia rebaudiana* Bertoni, NMR spectroscopy, biosynthetic pathway, isolation, characterization

## Abstract

A novel steviol glycoside, Rebaudioside *D17*, was identified from the leaf extract of *Stevia rebaudiana* Bertoni. This compound features a rare β-1→4 glycosidic linkage between two glucose units at the C19 position, distinguishing it from its structural isomer, Rebaudioside *D*. The aim of this study was to isolate and characterize Rebaudioside *D17* and investigate its biosynthetic origin. The compound was isolated and structurally characterized using comprehensive NMR spectroscopy including ^1^H, ^13^C, COSY, NOESY, Heteronuclear Single Quantum Coherence–Distortionless Enhancement by Polarization Transfer (HSQC-DEPT), Heteronuclear Multiple Bond Correlation (HMBC), Heteronuclear Single Quantum Coherence–Total Correlated Spectroscopy (HSQC-TOCSY), along with mass spectrometry analysis. A tentative biosynthetic pathway is proposed, involving Rebaudioside *E19*, a putative intermediate bearing the same β-1→4 glycosidic linkage at C19. Rebaudioside *E19* may serve as a common precursor to both Rebaudioside *D17* and Rebaudioside *U3*, a minor steviol glycoside previously reported in *Stevia rebaudiana* leaf extract, which also contains the same β-1→4 glycosidic linkage. The discovery of Rebaudioside *D17* expands the known diversity of steviol glycosides and provides new insights into glycosylation patterns in *Stevia rebaudiana*, which may support the development and production of novel sweeteners with improved sensory and physicochemical properties.

## 1. Introduction

*Stevia rebaudiana* Bertoni, a perennial shrub belonging to the Asteraceae (Compositae) family, is native to specific regions of South America. This plant has garnered significant attention due to its unique ability to biosynthesize intensely sweet compounds known as steviol glycosides, which are not found in other members of the *Stevia* genus. Among the approximately 230 species in the *Stevia* genus, only *S. rebaudiana* is known to produce steviol glycosides with high sweetness intensity [1,2].

For centuries, its leaf extracts have been traditionally used in Paraguay and Brazil to sweeten local teas and medicinal preparations [3,4]. The plant’s traditional use laid the foundation for its modern-day commercial cultivation and global popularity as a natural alternative to synthetic sweeteners, especially in light of growing health concerns related to obesity, diabetes, and metabolic syndrome [2].

The leaves of *S. rebaudiana* contain a diverse range of naturally sweet steviol glycosides, which are glycosylated derivatives of the diterpenoid aglycone steviol. These compounds, synthesized via the methylerythritol phosphate (MEP) pathway, can be up to 300 times sweeter than sucrose and are the primary contributors to the high-intensity sweetness of stevia extracts [5,6,7,8,9,10,11,12,13,14,15,16,17,18]. Steviol glycosides have been extensively studied for their potential to serve as natural high-potency sweeteners, offering sweetness with little to no caloric content [9,10,11,12,13,14,15,16]. The type and position of glycosidic linkages significantly influence the sweetness intensity, solubility, and sensory attributes of steviol glycosides, including their bitterness, onset, and lingering effects [16].

Structurally, they share a common steviol backbone, differing by the number and specific arrangement of sugar residues at the C13 and C19 positions. Commonly identified steviol glycosides include stevioside, Rebaudioside *A* through *O*, dulcoside *A*, steviolbioside and rubusoside, most of which feature β-1→2 and β-1→3 glycosidic linkages [2,17]. The structural diversity is largely attributed to the activity of UDP-dependent glycosyltransferases (UGTs), which catalyze the transfer of sugar residues to the steviol backbone in a regio- and stereo-specific manner [2].

Despite extensive research, the discovery of trace steviol glycosides remains challenging due to their low natural abundance and structural complexity. These compounds often exist in minute quantities within the leaf matrix, requiring highly sensitive and selective analytical tools for detection and characterization.

The identification of novel steviol glycosides contributes to a deeper understanding of the biosynthetic capabilities of *S. rebaudiana* and the enzymatic processes involved in glycosylation of steviol. The findings may also support future efforts in biotechnological production and diversification of natural sweeteners.

In this study, we report the isolation and structural characterization of a previously unreported steviol glycoside, Rebaudioside *D17*, from the leaf extract of *S. rebaudiana*. This novel compound features a rare β-1→4 glycosidic linkage at the C19 position and has a molecular weight of 1129.1534 g/mol. The synthetic anomer of Rebaudioside *D17*, known as Rebaudioside *A1G*, with an α-1→4 glycosidic linkage at the C19, was previously synthesized via enzymatic modification using cyclodextrin glycosyltransferase (CGTase) and was reported to exhibit improved sensory properties, including reduced bitterness and enhanced sweetness, compared to Rebaudioside *D*, which also contains five sugar moieties [18]. Given these reported sensory benefits, its natural β-anomer, Rebaudioside *D17*, warrants further investigation for potential sensory and functional properties.

Interestingly, Rebaudioside *U3*, a minor steviol glycoside previously identified in *S. rebaudiana,* also contains a β-1→4 glycosidic linkage at C19 and is currently the only steviol glycoside with this bond reported in the FAO JECFA 2023 Steviol Glycosides monograph [17]. Based on this shared structural feature, we propose a tentative biosynthetic pathway involving Rebaudioside *E19,* a putative intermediate featuring the same β-1→4 linkage at C19, as a possible precursor to Rebaudioside *D17* and Rebaudioside *U3.* Herein, we describe the isolation, structural elucidation, and proposed biosynthetic pathway for Rebaudioside *D17*.

## 2. Materials and Methods

### 2.1. General Experimental Procedures for Rebaudioside D17 (**1**)

#### 2.1.1. Isolation and Purification

Isolation of Rebaudioside *D17* (**1**) by HPLC was divided into two chromatographic steps. The first step involved purification using Preparative High-Performance Liquid Chromatography: Agilent 1200 Series Preparative LC System (Agilent Technologies, Waldbronn, Germany) equipped with preparative pump, autosampler, diode array detector and fraction collector with the following condition: Column: Agilent Zorbax SB-C18, 9.4 × 250 mm, 5 μm; Isocratic Mobile Phase: 25% MeCN in water; Flow Rate: 5 mL/min; Injection load: 100 µL of 100 mg/mL solution. The detection was by UV (210 nm). The fraction of interest was collected over multiple runs and concentrated by evaporation under reduced pressure.

The secondary purification was carried out using an Agilent 1100 Series HPLC system (Agilent Technologies, Waldbronn, Germany) equipped with a quaternary pump, autosampler, column compartment, multiple-wavelength detector, and analytical fraction collector with the following conditions: Column: Agilent Poroshell 120 SB-C18 2.7 µm, 4.6 × 150 mm; Column Temp: 40 °C; Isocratic Mobile Phase: 30% Acetonitrile (MeCN) in water; Flow Rate: 0.8 mL/min; Injection load: 2 µL of 50 mg/mL solution. The fraction of interest was collected over multiple runs and concentrated by evaporation under reduced pressure. The final purification was performed using the same column and conditions above, but injection load was 2 µL of 50 mg/mL solution. The fraction of interest was collected over multiple runs and dried by lyophilization under reduced pressure.

The stevia extract and final purified fraction of interest were analyzed using an Agilent 1200 Series HPLC system equipped with binary pump, autosampler, thermostat column compartment, diode array detector and an Agilent 6110 Single Quadrupole LC/MS systems operating in negative ion mode with the conditions summarized below. Column: Agilent Poroshell 120 SB-C18 2.7 µm, 4.6 × 150 mm; Column Temp: 40 °C; Mobile Phase A: Water (0.1% HCOOH) and Mobile Phase B: Acetonitrile (0.1% HCOOH) in an isocratic elution using 68% of mobile phase A and 32% of mobile phase B; Flow Rate: 0.5 mL/min; Injection volume: 2 μL. The detection was by UV (210 nm) and MSD (Scan Mode, API-ES), negative polarity, range 500–1500 Da). The collected yield was 16.3 mg and 94.5% purity.

#### 2.1.2. Mass Spectroscopy

The API-ES mass spectra and MS data were generated by an Agilent 6110 Single Quadrupole LC/MS spectrometer (Agilent Technologies, Little Falls, DE, USA) equipped with an atmospheric pressure ionization-electrospray ion source. Samples were analyzed by negative API-ES. Samples were prepared in a solvent mixture of H_2_O:MeCN (7:3), with a final concentration of approximately 1.0 mg/mL.

#### 2.1.3. Nuclear Magnetic Resonance

A sample of Rebaudioside *D17* (**1**) was prepared by dissolving 10 mg in 200 μL of pyridine-d_5_. The ^1^H, ^13^C, ^1^H–^1^H COSY, ^1^H–^13^C HSQC-DEPT, ^1^H–^13^C HMBC, ^1^H–^13^C HSQC-TOCSY and ^1^H–^1^H NOESY spectra were acquired on a Bruker 400 MHz spectrometer (Bruker BioSpin GmbH, Rheinstetten, Germany). NMR data were processed using MestReNova software (version 16.0.0-39276, Mestrelab Research S.L., Santiago de Compostela, Spain). Chemical shifts were referenced to pyridine-d_5_ (δ_H_ 7.19, 7.55, 8.71 ppm; δ_C_ 123.5, 135.5, 149.9 ppm).

### 2.2. Material Sources

Rebaudioside *D17* (**1**) was isolated from commercial *S. rebaudiana* extract (from stevia cultivar: PCS-13), sourced from PureCircle Sdn. Bhd., Bandar Enstek, Negeri Sembilan, Malaysia.

## 3. Results

### Rebaudioside D17

This work represents, to our knowledge, the first comprehensive report on the isolation and complete characterization of Rebaudioside *D17* (**1**). HPLC analysis of the stevia extract indicated that Compound **1** is present in a quantity of approximately 0.3% in the stevia extract on a dry weight basis (see Supplementary Figure S1). Compound **1** was isolated from *S. rebaudiana* extract using a two-step purification strategy involving preparative HPLC described above. The final purified compound was obtained as a white powder (94.5% chromatographic purity), with a sharp peak observed in the HPLC chromatogram at 7.853 min (See Supplementary Figure S2).

**Figure 1:**
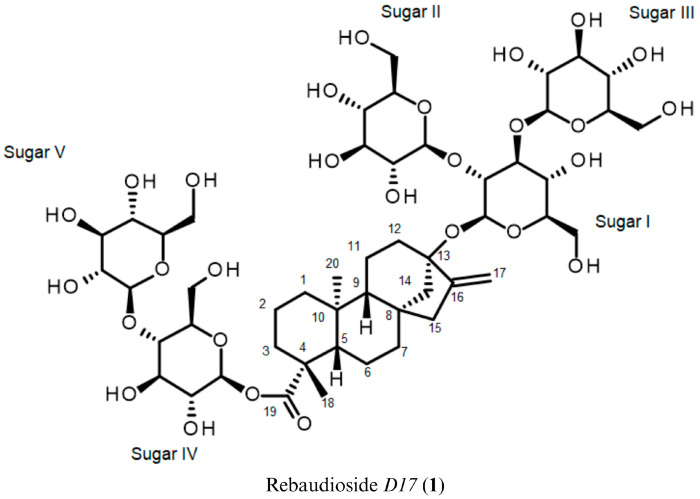
Chemical structure of Rebaudioside *D17* (**1**) with atom numbering for the aglycone and labelled sugar moieties (I–V).

LC-MS in negative ion mode (API-ES) revealed a deprotonated molecular ion at m/z 1127.4 [M-H]^−^ (see Supplementary Figure S3). This corresponds to a neutral molecular weight of 1128.4 g/mol, which is consistent with a steviol glycoside bearing five hexose units (calculated molecular weight = 1129.2 g/mol). The structure was elucidated using a comprehensive set of NMR techniques, including ^1^H, ^13^C, ^1^H–^1^H COSY, ^1^H–^1^H NOESY, Heteronuclear Single Quantum Coherence–Distortionless Enhancement Polarization Transfer (^1^H–^13^C HSQC-DEPT), Heteronuclear Multiple Bond Correlation (^1^H–^13^C HMBC) and Heteronuclear Single Quantum Coherence–Total Correlated Spectroscopy (^1^H–^13^C HSQC-TOCSY), with the spectra provided in the Supplementary Material (see Supplementary Figures S4–S11). Figure 1 illustrates the chemical structure of Rebaudioside *D17* (**1**).

Combined analysis of mass spectrometry and NMR spectra confirmed that compound **1** is a glycoside bearing a central diterpenoid core. The HSQC-DEPT spectrum revealed two methyl groups, which appeared as singlets at δ_H_ 1.20 and 1.24 ppm in the ^1^H NMR spectrum. Additionally, a signal at δ_C_ 104.4 ppm demonstrated the presence of an exo-methylene carbon. This assignment was further supported by the correlations of two olefinic protons at δ_H_ 4.98 and 5.62 ppm with the δ_C_ 104.4 ppm signal, confirming the presence of an exocyclic double bond. Nine methylene and two methine protons were observed between δ_H_ 0.72–2.62 ppm, which is characteristic of the *ent*-kaurane diterpenoid skeleton previously reported in Stevia extracts [5]. The presence of the *ent*-kaurane diterpenoid aglycone was supported by ^1^H–^1^H COSY correlations of H-1/H-2; H-2/H-3; H-5/H-6; H-6/H-7; H-9/H-11; H-11/H-12 and ^1^H–^13^C HMBCs of H-14/C-15, C-16; H-17/C-13, C-15; H-18/C-3, C-5, C-19 and H-20/C-5, C-9 (See Supplementary Table S1 for complete ^1^H and ^13^C assignments of the *ent*-kaurane diterpenoid).

Correlations observed in the ^1^H–^1^H NOESY spectrum were used to assign the relative stereochemistry of the central diterpene core. The NOE correlations between H-14 and H-20 indicated that these protons are located on the same face of the molecule. Due to signal overlap between H-3 and H-5, NOE correlations between H-5 and H-9 could not be confirmed. However, the absence of NOE correlations between H-14 and H-9, as well as H-20 and H-5, suggests that H-9 and H-5 are positioned on the opposite face relative to H-14 and H-20. The application of ^1^H–^1^H NOESY thus confirmed the relative stereochemistry of the aglycone, further supporting its identity as an *ent*-kaurane diterpenoid consistent with steviol, the known aglycone of steviol glycosides in *S. rebaudiana* [5].

Following the identification of steviol as the aglycone (molecular weight = 318.46 g/mol), Compound **1** was hypothesized to be a steviol glycoside with five additional hexose sugar moieties, based on its molecular weight of 1129.1534 g/mol as determined by LC-MS analysis.

The presence of five anomeric protons, evident from ^1^H and ^1^H–^13^C HSQC-DEPT spectra, confirmed the presence of five sugar units in the structure. Doublets with large coupling constants were observed for four of the anomeric protons at the δ_H_ 5.53 (*J* = 8.0 Hz), 5.32 (*J* = 8.0 Hz), 6.01 (*J* = 8.0 Hz) and 5.20 ppm (*J* = 8.0 Hz), consistent with β-orientation. The anomeric proton at δ_H_ 5.03 ppm was partially overlapped with the residual water signal at δ_H_ 5.02 ppm but its apparent doublet pattern with a coupling constant of *J* = 8.0 Hz remained discernible, which also suggests β-configuration, as confirmed by both ^1^H and ^1^H–^13^C HSQC-DEPT spectra.

The complete assembly of each sugar moiety was elucidated based on the correlations observed in the ^1^H–^1^H COSY and ^1^H–^13^C HSQC-TOCSY spectra, combined with ^1^H–^1^H NOESY data that revealed the stereochemistry of each unit. For sugar I, the β-orientation of the anomeric proton indicated that both H-1^i^ and H-2^i^ were axial. NOESY correlations from H-1^i^ to both H-3^i^ and H-5^i^ indicated that both H-3^i^ and H-5^i^ were also axial, consistent with a β-D-glucopyranose configuration (see Supplementary Figure S11). Similar analysis was performed for the remaining sugar units, all of which were likewise confirmed to be β-D-glucoses.

Long-range ^1^H–^13^C correlations observed in the HMBC experiment from the anomeric proton at δ_H_ 5.03 ppm to a quaternary carbon at δ_C_ 86.3 ppm (C-13) allowed it to be assigned as the anomeric proton (H-1^i^) of sugar I. Likewise, the anomeric proton of sugar IV was assigned based on the HMBC from the anomeric proton at δ_H_ 6.01 ppm to a carbonyl carbon at δ_C_ 176.8 ppm.

Further analysis of the 1D and 2D NMR data allowed the assignment of the remaining protons in both sugar I and sugar IV. The relatively downfield chemical shift of C-2 and C-3 (δ_C_ 80.7 and 87.9 ppm) in sugar I suggested a 2,3-branched-D-glucotriosyl substituent at C-13, which was further confirmed by HMBCs. The 1→2 sugar linkage between sugar I and sugar II was confirmed by the HMBC observed from the anomeric protons at δ_H_ 5.53 ppm to the carbon at δ_C_ 80.7 ppm. Similarly, the 1→3 sugar linkage between sugar I and sugar III was confirmed by the HMBC from the anomeric proton at δ_H_ 5.32 ppm to the carbon at δ_C_ 87.9 ppm.

**Figure 2:**
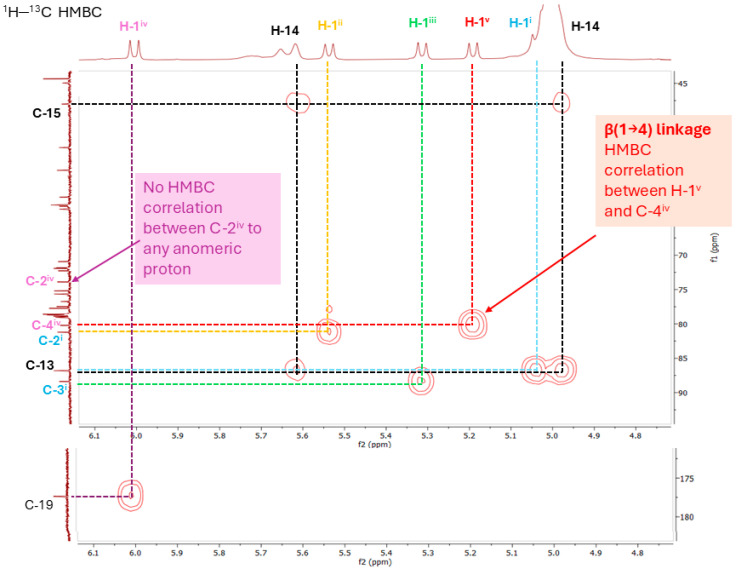
Annotated HMBC spectrum of Rebaudioside *D17* (**1**) showing key long-range ^1^H–^13^C correlations used to establish glycosidic linkages. Sugar moieties (I–V) are colour-coded, and corresponding ^1^H and ^13^C labels share the same colour for clarity: Sugar I (blue), Sugar II (orange), Sugar III (green), Sugar IV (purple) and Sugar V (red). Dashed lines indicate correlations confirming linkage positions and branching points, with each line matching the colour of the originating proton. The correlation between H-1^v^ and C-4^iv^ (highlighted in red) confirms a β-1→4 glycosidic linkage. No correlation between C-2^iv^ and any anomeric proton was observed, excluding a β-1→2 glycosidic linkage at C-2^iv^.

The two glucose moieties on the C19 glucosyl group of steviol were assigned in a similar manner. The relatively downfield chemical shift of C-4 (δ_C_ 79.6 ppm) in sugar IV suggested a β-D-glucopyranosyl-(1→4)-D-glucopyranosyl substituent at C-19. The presence of a 1→4 sugar linkage between sugar IV and sugar V was confirmed by the HMBC from the anomeric proton at δ_H_ 5.20 ppm to the carbon at δ_C_ 79.6 ppm. The key HMBCs supporting the β-1→4 glycosidic linkage are illustrated and annotated in Figure 2, with the full HMBC spectrum provided in Supplementary Material (see Supplementary Figure S9). Notably, no correlation between C-2^iv^ and any anomeric proton was observed, excluding the possibility of a β-1→2 glycosidic linkage at C-2^iv^, as would be expected for Rebaudioside *D* [5].

The ^1^H and ^13^C chemical shifts for the glycosides at C-13 and C-19 are summarized in Supplementary Table S2. The structure of Rebaudioside *D17* (**1**), containing a relatively rare 1→4 sugar linkage was established as (13-[(2-O-β-D-glucopyranosyl-3-O-β-D-glucopyranosyl-β-D-glucopyranosyl)oxy] *ent*-kaur-16-en-19-oic acid-[(4-O-β-D-glucopyranosyl-β-D-glucopyranosyl) ester]).

The structural elucidation of Rebaudioside *D17* (**1**) highlights the power of advanced NMR techniques in resolving complex glycosidic architectures. HSQC-TOCSY, NOESY, and HMBC experiments were used in combination to support the assignment of sugar linkages and stereochemistry, despite significant signal overlap in the carbohydrate region. The identification of a rare β-1→4 glycosidic linkage at the C-19 position distinguishes Rebaudioside *D17* (**1**) from other known steviol glycosides, which typically feature β-1→2 or β-1→3 linkages. This unique structural feature may influence molecular conformation and taste receptor interactions, suggesting potential implications for sweetness perception and enzymatic biosynthesis, warranting further investigation into its functional and sensory properties. The presence of this linkage also suggests the involvement of a distinct glycosyltransferase with unusual regioselectivity, which warrants further enzymatic and pathway investigation.

## 4. Discussion

### 4.1. Proposed Biosynthetic Pathway of Rebaudioside D17 (**1**)

Following the structural elucidation of the novel compound Rebaudioside *D17* (**1**), we investigated its possible biosynthetic origin in *S. rebaudiana*. Structurally, Rebaudioside *D17* (**1**) resembles another well known steviol glycoside, Rebaudioside *D*, but features a relatively rare β-1→4 glycosidic linkage between two glucose units at the C19 position, in contrast to the more common β-1→2 glycosidic linkage observed in Rebaudioside *D*. This unusual linkage is of particular interest, as it deviates from the predominant glycosylation patterns observed in steviol glycosides and may influence the compound’s sweetness intensity, solubility, and sensory attributes [18]. The presence of this unusual linkage prompted us to hypothesize possible biosynthetic pathways that could account for the formation of Rebaudioside *D17* (**1**).

Given the structural similarity, Rebaudioside *A*—the known precursor of Rebaudioside *D*—was initially considered as potential precursor. Rebaudioside *A* undergoes further glycosylation at the C13 position to yield Rebaudioside *D* via the action of specific UDP–glucosyltransferases (UGTs), such as UGT91D2, which catalyze β-1→2 glycosidic linkages [19]. A similar glycosylation event might have occurred during the biosynthesis of Rebaudioside *D17* (**1**), though involving the formation of a β-1→4 glycosidic bond at the C19 position, potentially mediated by either UGT exhibiting broader specificity or a different UGT with distinct regioselectivity. Although Rebaudioside *D17* (**1**) has not previously been identified as a glycosylation product of Rebaudioside *A*, the diversity of UDP–glucosyltransferases (UGTs) expressed in *S. rebaudiana* allows for the possibility of minor biosynthetic pathways that could give rise to less common steviol glycosides [2,6].

The presence of Rebaudioside *U3* (**2**), the only steviol glycoside with β-1→4 glycosidic linkage at the C19 reported in the FAO JECFA 2023 Steviol Glycosides monograph, led us to hypothesize an alternative biosynthetic route [17,20]. First reported in 2018, Rebaudioside *U3* (**2**) contains a β-1→4 glycosidic linkage with glucose and a β-1→2 glycosidic linkage with xylose at the C19 position, forming a branched trisaccharide structure [20]. Additionally, it features a β-1→2 glycosidic linkage with a glucose at the C13 position. Given the shared β-1→4 glycosidic linkage at the C19, we propose that both Rebaudioside *D17* (**1**) and Rebaudioside *U3* (**2**) may originate from a common putative biosynthetic precursor—we tentatively named Rebaudioside *E19* (**3**)—bearing a β-1→4 glycosidic linkage with glucose at C19 and a β-1→2 glycosidic linkage with glucose at C13.

**Figure 3:**
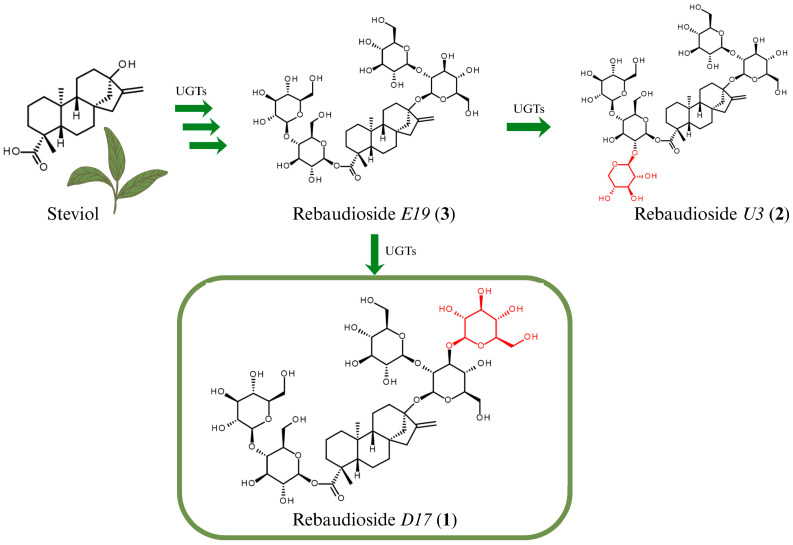
Proposed biosynthetic pathway for Rebaudioside *D17* (**1**) and Rebaudioside *U3* (**2**) from steviol via the putative intermediate Rebaudioside *E19* (**3**), highlighting in red the addition of a β-1→3-linked glucose at C13 for Rebaudioside *D17* (**1**) and a β-1→2-linked xylose at C19 for Rebaudioside *U3* (**2**).

From this intermediate, Rebaudioside *D17* (**1**) could be formed via additional β-1→3 glucosylation at C13, while Rebaudioside *U3* (**2**) could arise via β-1→2 xylosylation at C19. While Rebaudioside *A* could theoretically serve as a precursor, the shared structural features between Rebaudioside *D17* (**1**) and Rebaudioside *U3* (**2**) suggest that Rebaudioside *E19* (**3**) is more likely to be the biosynthetic precursor. A plausible pathway for the biosynthesis of both Rebaudioside *D17* (**1**) and Rebaudioside *U3* (**2**) via their common putative precursor, Rebaudioside *E19* (**3**), is illustrated in Figure 3. The proposed pathway is supported by the structural similarity between Rebaudioside *D17* (**1**) and Rebaudioside *U3* (**2**), particularly the shared β-1→4 linkage at C19, and is consistent with the known diversity and substrate flexibility of UDP–glycosyltransferases (UGTs) in *S. rebaudiana* [2,19,21,22].

To validate the proposed biosynthetic pathway, future studies should focus on identifying and characterizing the UGTs responsible for the β-1→4 glycosylation. This could be achieved, for example, through transcriptomic profiling of *S. rebaudiana* tissues enriched in Rebaudioside *D17* (**1**), followed by heterologous expression and in vitro enzyme assays using candidate UGTs and relevant substrates. Such insights will contribute to a deeper understanding of steviol glycoside biosynthesis and the enzymatic mechanisms underlying their structural diversity.

### 4.2. Potential Influence of Glycosidic Linkage on Sensory and Physicochemical Properties

Minor steviol glycosides such as Rebaudioside *D17* (**1**) may exhibit distinct sensory and physicochemical advantages. Understanding the properties of Rebaudioside *D17* (**1**) could clarify the structure–function effect of glycosidic linkage on both sensory and physicochemical properties of steviol glycosides, especially when the molecules have the same type and number of glucose moieties, such as Rebaudioside *D* and Rebaudioside *AM*. These insights not only serve as valuable guidance for predicting the properties of other steviol glycoside sweeteners but also deepen our understanding of structure–function relationships in high-potency sweeteners.

Numerous studies and publications have explored the influence of sugar moiety number and glycosidic linkage position on the physicochemical and sensory properties of steviol glycosides. Among these, our earlier publication provides specific data demonstrating how structural variations in glycosylation patterns affect attributes such as sweetness perception and solubility [23]. Table 1 summarizes the glycosidic linkages of the tested steviol glycosides—Rebaudioside *D*, Rebaudioside *M* and Rebaudioside *AM*—alongside their reported sweetness perception threshold concentration and maximum soluble concentration.

Based on the data in Table 1, the relationship between total glucose moieties and glycosidic linkage with sensory and physicochemical attributes such as sweetness perception and solubility is not immediately apparent [23]. Although Rebaudioside *M*, which has the highest number of glucose moieties (six), exhibits the lowest sweetness perception threshold concentration (24 ppm), Rebaudioside *A*, with only four glucose moieties, shows the second-lowest threshold (30 ppm). Despite having the same number of glucose moieties (five), the sweetness perception threshold concentration of Rebaudioside *D* and Rebaudioside *AM* differ significantly (32.5 ppm Vs. 50 ppm, respectively), signifying that the total glucose count is not the sole factor affecting sweetness perception.

Additionally, in a separate publication filed by Xiao et al., the α-anomer of Rebaudioside *D17* (**1**), known as Rebaudioside *A1G*, with an α-1→4 glycosidic linkage at the C19, was reported to exhibit improved sensory properties, including reduced bitterness and enhanced sweetness, compared to Rebaudioside *D*, which also contains five glucose moieties [18]. The reported sensory benefits further support the hypothesis that glycosidic linkage type plays a critical role in determining sensory attributes and underscore the need to investigate the natural β-anomer, Rebaudioside *D17* (**1**), for potential sensory and functional properties.

Numerous studies have investigated the structure–function relationship in sweetness using two main approaches: structure-based studies that apply cheminformatics, Quantitative Structure–Activity Relationship (QSAR) and machine learning to correlate molecular descriptors with sweetness, and receptor-focused studies that employ homology modelling and molecular docking to examine ligand–receptor interactions and binding site preferences [24,25,26,27,28]. For sweetness perception, structural variation may affect how the molecules interact with human sweetness receptors (T1R2/T1R3). A previous study by Hao et al. reported molecular docking and binding experiments demonstrating that structurally distinct steviol glycosides interact with the T1R2/T1R3 sweet taste receptor at multiple binding sites and orientations, highlighting the influence of molecular structure in receptor binding and the resulting sweetness profile [29]. Recent cryo-electron microscopy studies on T1R2/T1R3 have provided high-resolution structures of the human sweet taste receptor, further advancing receptor-focused investigations into sweetness perception [30].

Similarly, the maximum soluble concentration of Rebaudioside *AM* (5%) is 50-fold higher compared to that of Rebaudioside *D* (0.1%), despite both having five glucose moieties. The significant difference between Rebaudioside *D* and Rebaudioside *AM* suggests that the glycosidic linkage may influence the spatial arrangement of sugar moieties, which in turn affects their hydration and ability to form hydrogen bonds with water.

Collectively, these observations indicate that physicochemical and sensory properties of steviol glycosides are not solely determined by the number of sugar moieties; glycosidic linkage patterns evidently play a critical role by influencing the three-dimensional structure of steviol glycosides. Evaluating Rebaudioside *D17* (**1**) will not only help determine its sensory profile but also contribute to a deeper understanding of structure function relationship, i.e., how linkage type influences the functional behaviour of steviol glycosides. Comparative sensory profiling with established steviol glycosides such as Rebaudioside *D* and Rebaudioside *M* will be essential to determine its relative performance.

## 5. Conclusions

We report the full isolation and spectral characterization of (13-[(2-O-β-D-glucopyranosyl-3-O-β-D-glucopyranosyl-β-D-glucopyranosyl)oxy] *ent*-kaur-16-en-19-oic acid-[(4-O-β-D-glucopyranosyl-β-D-glucopyranosyl) ester]), Rebaudioside *D17* (**1**), a novel steviol glycoside featuring a rare β-1→4 glycosidic linkage between two glucose units at the C19 position. Rebaudioside *D17* (**1**) was structurally elucidated using comprehensive 1D and 2D NMR techniques, including ^1^H, ^13^C, ^1^H–^1^H COSY, ^1^H–^13^C HSQC-DEPT, ^1^H–^13^C HMBC, ^1^H–^13^C HSQC-TOCSY and ^1^H–^1^H NOESY, which enabled detailed assignment of its glycosidic architecture. Based on its structural features and comparison with known steviol glycosides, we propose a plausible biosynthetic pathway of Rebaudioside *D17* (**1**) involving a putative intermediate, Rebaudioside *E19* (**3**), which may also give rise to Rebaudioside *U3* (**2**), a known steviol glycoside present in *S. rebaudiana*. The shared β-1→4 linkage at C19 in both compounds supports the hypothesis of a minor biosynthetic route facilitated by the substrate flexibility of UDP–glucosyltransferases (UGTs) in *S. rebaudiana*.

Future work is needed to evaluate the sensory properties of Rebaudioside *D17*. Given its low abundance, future efforts will focus on synthetic approaches to produce sufficient quantities. Advances in biotransformation, synthetic biology and metabolic engineering offer promising strategies to design microbial platforms capable of expressing specific UGTs and optimizing glycosylation pathways. These approaches enable targeted and scalable biosynthesis, offering a sustainable alternative to plant extraction.

Preliminary structural insights suggest that the unique β-1→4 linkage may influence the compound’s attributes, such as bitterness, sweetness, lingering and solubility. Literature has shown that variations in glycosidic linkage can significantly impact these sensory and physicochemical properties.

The discovery of Rebaudioside *D17* (**1**) expands the known diversity of steviol glycosides and highlights the importance of continued exploration of glycosylation patterns in *S. rebaudiana*. Understanding how structural variations influence sweetness and physicochemical properties may support the development of next-generation sweeteners with improved taste profiles.

## Figures and Tables

**Table 1:** Total glucose moieties, sweetness perception threshold concentration (ppm) and maximum soluble concentration (%) of Rebaudioside *A*, Rebaudioside *D*, Rebaudioside *M* and Rebaudioside *AM* [23].

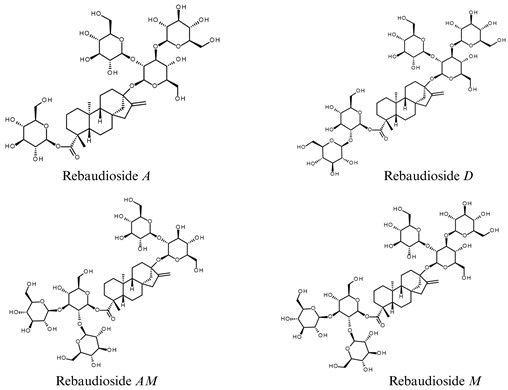			
Steviol Glycoside	Total Glucose Moieties	Sweetness Perception Threshold Concentration (ppm) *	Maximum Soluble Concentration (%) **
Rebaudioside *A*	4	30.0	NA
Rebaudioside *D*	5	32.5	0.1%
Rebaudioside *AM*	5	50.0	5%
Rebaudioside *M*	6	24.0	0.1%

* Sweetness perception threshold concentrations represent the lowest concentration at which sweetness was perceptible in water. ** Maximum soluble concentration (%) refers to the highest tested concentration that remained clear after 48 h.

## Data Availability

The original contributions presented in this study are included in the article/Supplementary Materials. Further inquiries can be directed to the corresponding author.

## Supplementary Materials

The following supporting information can be downloaded at https://www.mdpi.com/article/10.3390/biom16010146/s1, Figure S1. HPLC chromatogram of *Stevia rebaudiana* extract highlighting the peak corresponding to Rebaudioside *D17*; Figure S2. HPLC chromatogram of Purified Rebaudioside *D17*; Figure S3. The mass spectrum of Rebaudioside *D17*; Figure S4. The ^1^H NMR spectrum of Rebaudioside *D17*; Figure S5. The ^13^C NMR spectrum of Rebaudioside *D17*; Figure S6. ^1^H–^1^H COSY NMR spectrum of Rebaudioside *D17* (Sugar region); Figure S7. ^1^H–^1^H COSY NMR spectrum of Rebaudioside *D17* (Sugar region and aglycone region); Figure S8. ^1^H–^13^C HSQC-DEPT NMR spectrum of Rebaudioside *D17* (Sugar region and aglycone region); Figure S9. ^1^H–^13^C HMBC NMR spectrum of Rebaudioside *D17* (Sugar region and aglycone regions); Figure S10. ^1^H–^13^C HSQC-TOCSY NMR spectrum of Rebaudioside *D17* (Sugar region); Figure S11. ^1^H–^1^H NOESY NMR spectrum of Rebaudioside *D17*; Table S1. ^1^H and ^13^C NMR chemical shift data for the aglycone of Rebaudioside *D17*; Table S2. ^1^H and ^13^C NMR chemical shift data for the sugar moieties of Rebaudioside *D17*.

## References

[1] Kinghorn A.D., Soejarto D.D., Wagner H., Hikino H., Farnsworth N.R. (1985). Current status of stevioside as a sweetening agent for human use. Economic and Medicinal Plant Research.

[2] Ceunen S., Geuns J.M.C. (2013). Steviol Glycosides: Chemical Diversity, Metabolism, and Function. J. Nat. Prod..

[3] Brandle J.E., Starratt A.N., Gijzen M. (1998). *Stevia rebaudiana*: Its agricultural, biological, and chemical properties. Can. J. Plant Sci..

[4] Lewis W.H. (1992). Early uses of *Stevia rebaudiana* (Asteraceae) leaves as a sweetener in Paraguay. Econ. Bot..

[5] Prakash I., Bunders C., Devkota K.P., Charan R.D., Ramirez C., Priedemann C., Markosyan A. (2014). Isolation and Characterization of a Novel Rebaudioside M Isomer from a Bioconversion Reaction of Rebaudioside A and NMR Comparison Studies of Rebaudioside M Isolated from *Stevia rebaudiana* Bertoni and *Stevia rebaudiana* Morita. Biomolecules.

[6] Brandle J., Telmer P. (2007). Steviol glycoside biosynthesis. Phytochemistry.

[7] Wölwer-Rieck U., May B., Lankes C., Wüst M. (2014). Methylerythritol and Mevalonate Pathway Contributions to Biosynthesis of Mono, Sesqui, and Diterpenes in Glandular Trichomes and Leaves of *Stevia rebaudiana* Bertoni. J. Agric. Food Chem..

[8] Bridel M., Lavielle R. (1931). The Sweet Principle of Kaahe-e (*Stevia rebaudiana*). Pharm. Chem. J..

[9] Prakash I., DuBois G.E., Clos J.F., Wilkens K.L., Fosdick L.E. (2008). Development of rebiana, a natural, non-caloric sweetener. Food Chem. Toxicol..

[10] Chaturvedula V.S.P., Prakash I. (2011). A new diterpene glycoside from *Stevia rebaudiana*. Molecules.

[11] Chaturvedula V.S.P., Prakash I. (2011). Structures of the novel diterpene glycosides from *Stevia rebaudiana*. Carbohydr. Res..

[12] Chaturvedula V.S.P., Rhea J., Milanowski D., Mocek U., Prakash I. (2011). Two minor diterpene glycosides from the leaves of *Stevia rebaudiana*. Nat. Prod. Commun..

[13] Chaturvedula V.S.P., Clos J.F., Rhea J., Milanowski D., Mocek U., DuBois G.E., Prakash I. (2011). Minor diterpenoid glycosides from the leaves of *Stevia rebaudiana*. Phytochem. Lett..

[14] Chaturvedula V.S.P., Mani U., Prakash I. (2011). Structures of the novel α-glucosyl linked diterpene glycosides from *Stevia rebaudiana*. Carbohydr. Res..

[15] Prakash I., Campbell M., San Miguel R.I., Chaturvedula V.S.P. (2012). Synthesis and sensory evaluation of *ent*-Kaurane diterpene glycosides. Molecules.

[16] Prakash I., Campbell M., San Miguel R.I., Chaturvedula V.S.P. (2013). Catalytic hydrogenation of the sweet principles of *Stevia rebaudiana*, rebaudiosde B, rebaudioside C and rebaudioside D and sensory evaluation of their reduced derivatives. Int. J. Mol. Sci..

[17] FAO/WHO (2023). Framework for steviol glycosides. Compendium of Food Additive Specifications.

[18] Xiao M., Xu L., Zhu L., Wang W., Peng P., Du G., Yue W. (2019). Stevioside Derivative Rebaudioside *A1G*, Preparation, Purification and Application Thereof. WIPO Patent.

[19] Olsson K., Carlsen S., Semmler A., Simón E., Mikkelsen M.D., Møller B.L. (2016). Microbial production of next-generation stevia sweeteners. Microb. Cell Fact..

[20] Purkayastha S., Markosyan A., Chow S.Y., Prakash I., Clos J., Peng I.X., Kagan M.Z., Sukits S.F., Somayajula K.V., Nizam bin Nawi K. (2019). Stevia-Derived Molecules, Methods of Obtaining such Molecules, and Uses of the Same. WIPO Patent.

[21] Kishore G., Motion M., Hicks P.M., Hansen J., Houghton-Larsen J., Hansen E.H., Mikkelsen M.D., Tavares S., Blom C. (2011). Recombinant Production of Steviol Glycosides. PCT International Publication.

[22] Biswas P., Kumari A., Modi A., Kumar N. (2024). Improvement and Regulation of Steviol Glycoside Biosynthesis in *Stevia rebaudiana* Bertoni. Gene.

[23] Markosyan A., Ramandach S., Afzaal Bin Hasim M., Nizam Bin Nawi K., Chow S.Y., Purkayastha S., Petit M. (2019). High-Purity Steviol Glycosides. WIPO Patent.

[24] Zheng S., Chang W., Xu W., Xu Y., Lin F. (2019). e-Sweet: A machine-learning based platform for the prediction of sweetener and its relative sweetness. Front. Chem..

[25] Lin F., Ji Y., Xu S. (2024). Sweetener identification using transfer learning and attention mechanism. CyTA-J. Food.

[26] Tuwani R., Wadhwa S., Bagler G. (2019). BitterSweet: Building machine learning models for predicting all bitter and sweet taste of small molecules. Sci. Rep..

[27] Tang N. (2023). Insights into chemical structure-based modeling for new sweetener discovery. Foods.

[28] Capela J., Correia J., Pereira V., Rocha M. (2022). Development of deep learning approaches to predict relationships between chemical structures and sweetness. Proceedings of the 2022 International Joint Conference on Neural Networks (IJCNN).

[29] Hao S., Guthrie B., Kim S., Balanda S., Kubicek J., Murtaza B., Khan N.A., Khakbaz P., Su J., Goddard III W.A. (2024). Steviol rebaudiosides bind to four different sites of the human sweet taste receptor (T1R2/T1R3) complex explaining confusing experiments. Commun. Chem..

[30] Shi Z., Xu W., Wu L., Yue X., Liu S., Ding W., Zhang J., Meng B., Zhao L., Liu X. (2025). Structural and functional characterization of human sweet taste receptor. Nature.

