# Supplementary material for: Discovery of Novel Minor Steviol Glycoside from the Stevia rebaudiana: Structural Characterization and Proposed Biosynthetic Pathway of Rebaudioside D17
Source: Biomolecules. 2026 Jan 14;16(1):146. doi: 10.3390/biom16010146 (PMC12838583; doi:10.3390/biom16010146)
Supplement: Supplementary file 1 [file biomolecules-16-00146-s001.zip › biomolecules-4015945-SI.pdf]

Supplementary Materials

Supplementary Figure S1

Figure S1. HPLC chromatogram of *Stevia rebaudiana* extract highlighting the peak corresponding to Rebaudioside *D17*.

The peak at a retention time of 7.853 minutes corresponds to Rebaudioside *D17*.

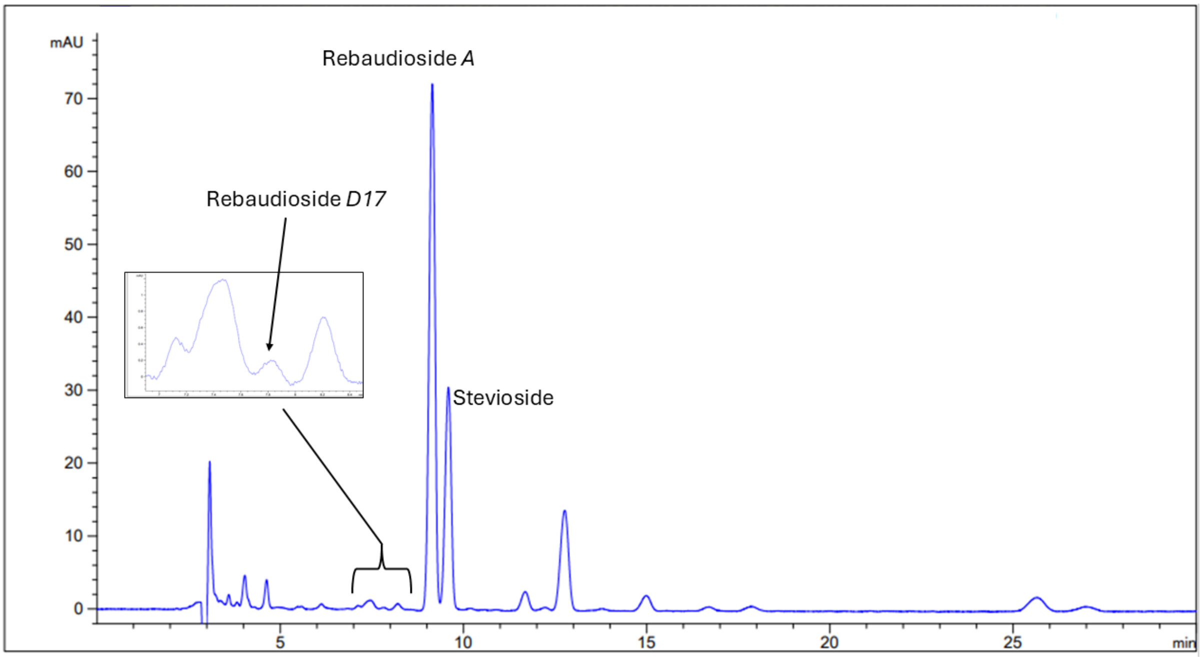

Supplementary Figure S2

Figure S2. HPLC chromatogram of Purified Rebaudioside *D17*.

A sharp peak at a retention time of 7.853 minutes corresponds to Rebaudioside *D17*.

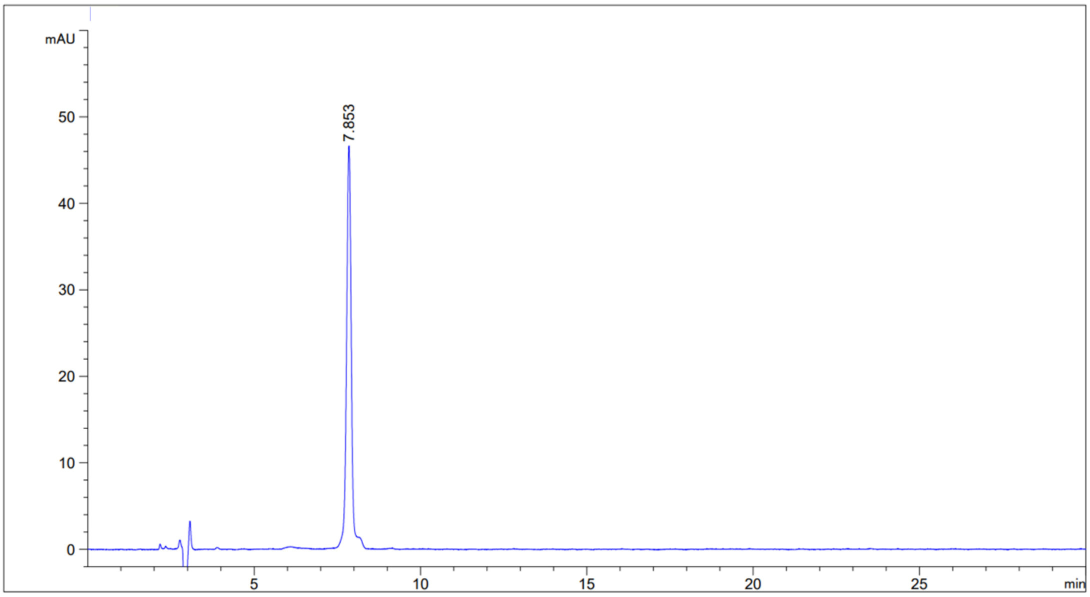

Supplementary Figure S3

Figure S3. The mass spectrum of Rebaudioside *D17*.

LC-MS in negative ion mode (API-ES) revealed a deprotonated molecular ion at m/z 1127.4 [M-H]$^-$, which corresponds to a neutral molecular weight of 1128.4 g/mol.

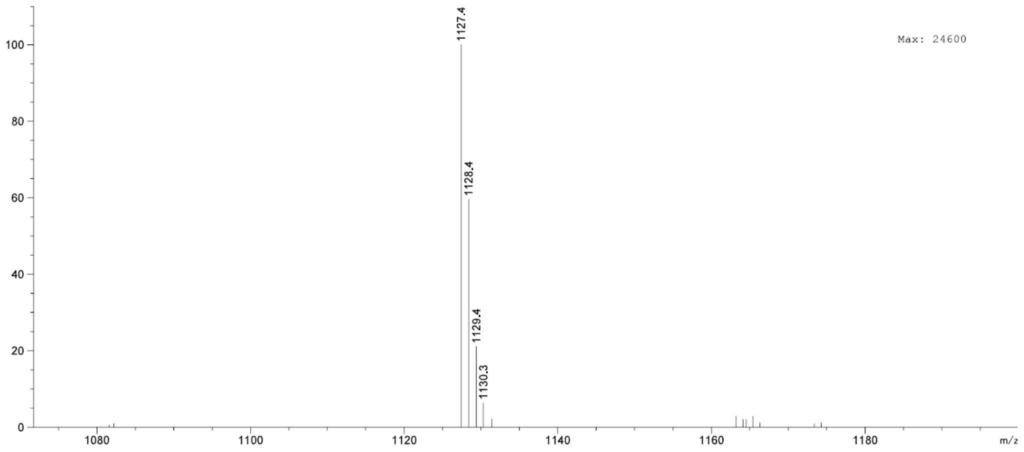

Supplementary Figure S4

Figure S4. The ^{1}H NMR spectrum of Rebaudioside *D17*.

Selected peaks are annotated to indicate key proton environments relevant for the structural assignment of β-1,4 glycosidic linkage. Full chemical shift assignments are provided in Supplementary Table S12 and Table S13.

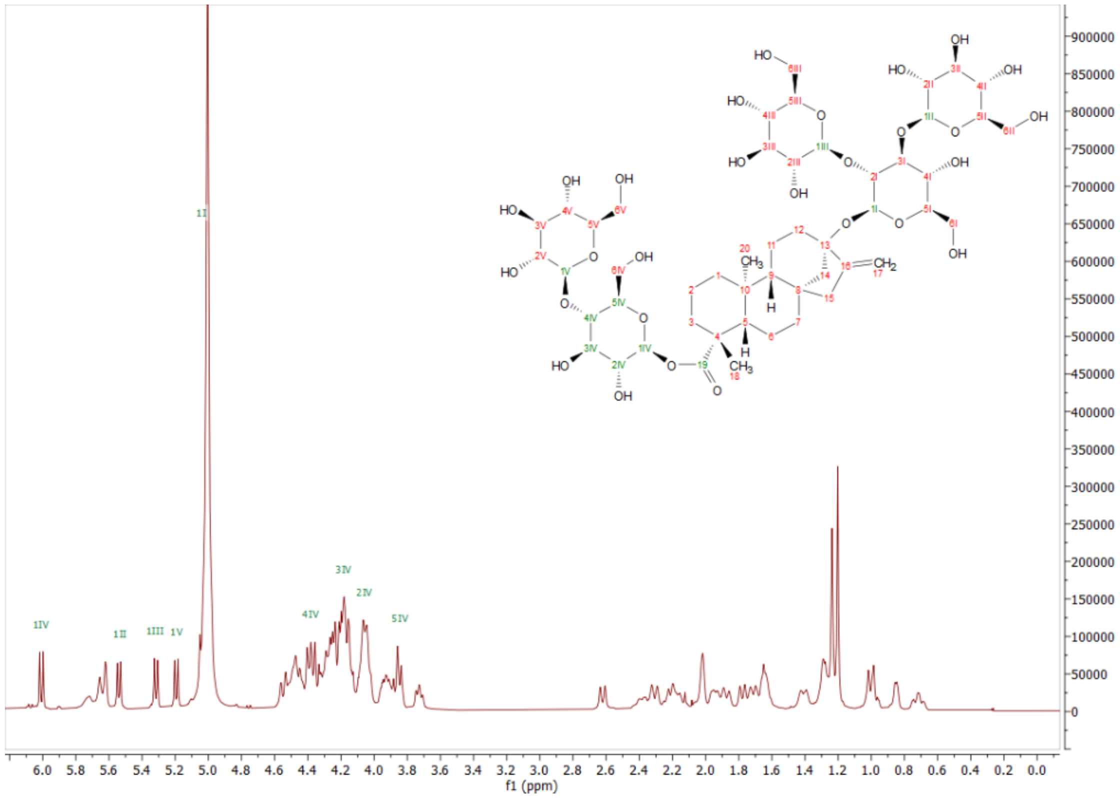

Supplementary Figure S5

Figure S5. The ^{13}C NMR spectrum of Rebaudioside *D17*.

Selected peaks are annotated to highlight key carbon environments relevant for the structural assignment of β-1,4 glycosidic linkage. Full chemical shift assignments are provided in Supplementary Table S12 and Table S13.

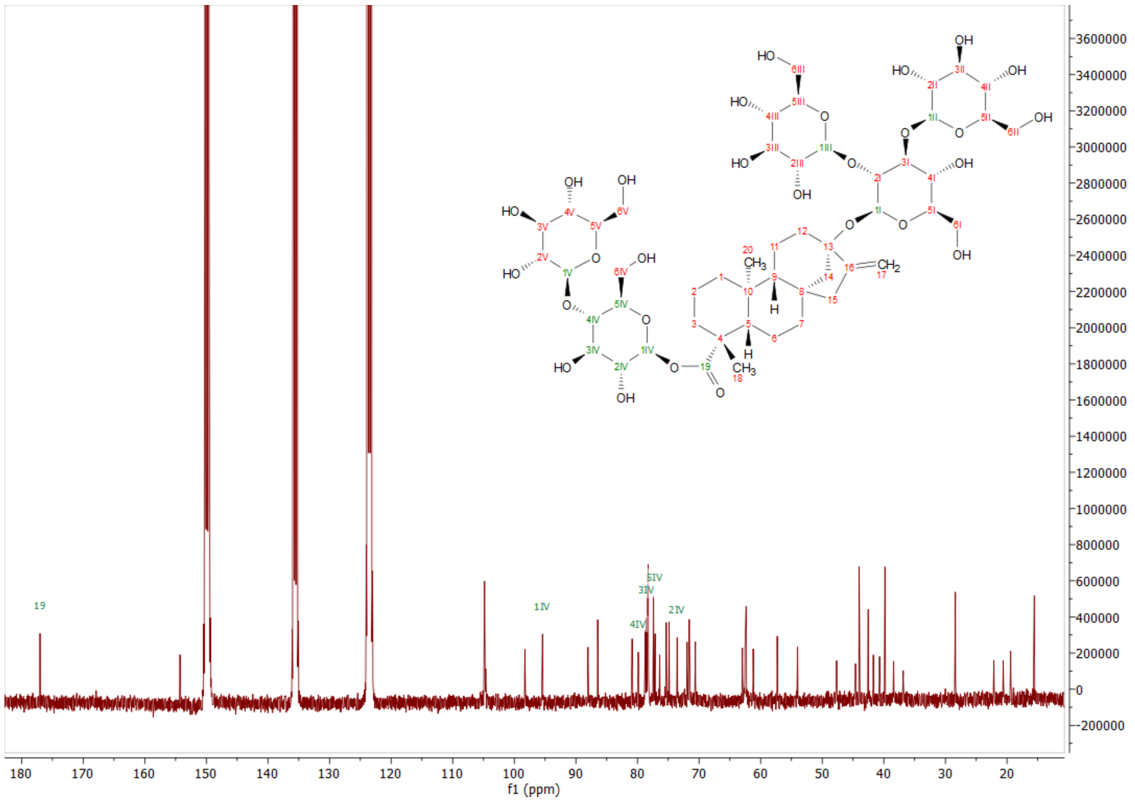

Supplementary Figure S6

Figure S6. ^1H–^1H COSY NMR spectrum of Rebaudioside *D17* (Sugar region).

Selected peaks are annotated to highlight key proton correlations relevant to the structural assignment of sugar IV.

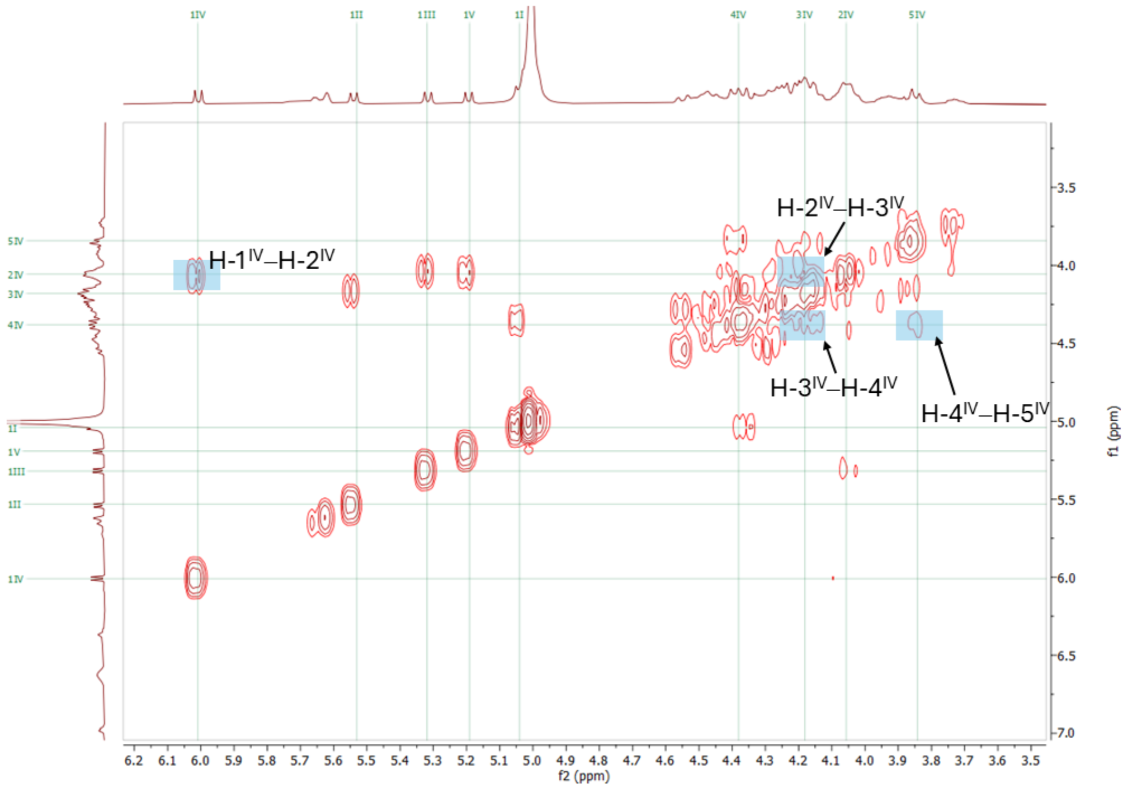

Supplementary Figure S7

Figure S7. ¹H–¹H COSY NMR spectrum of Rebaudioside *D17* (Sugar region and aglycone region).

The spectrum includes both sugar and aglycone regions of Rebaudioside *D17*. Selected cross-peaks are annotated to highlight key proton correlations relevant to the structural assignment of sugar IV.

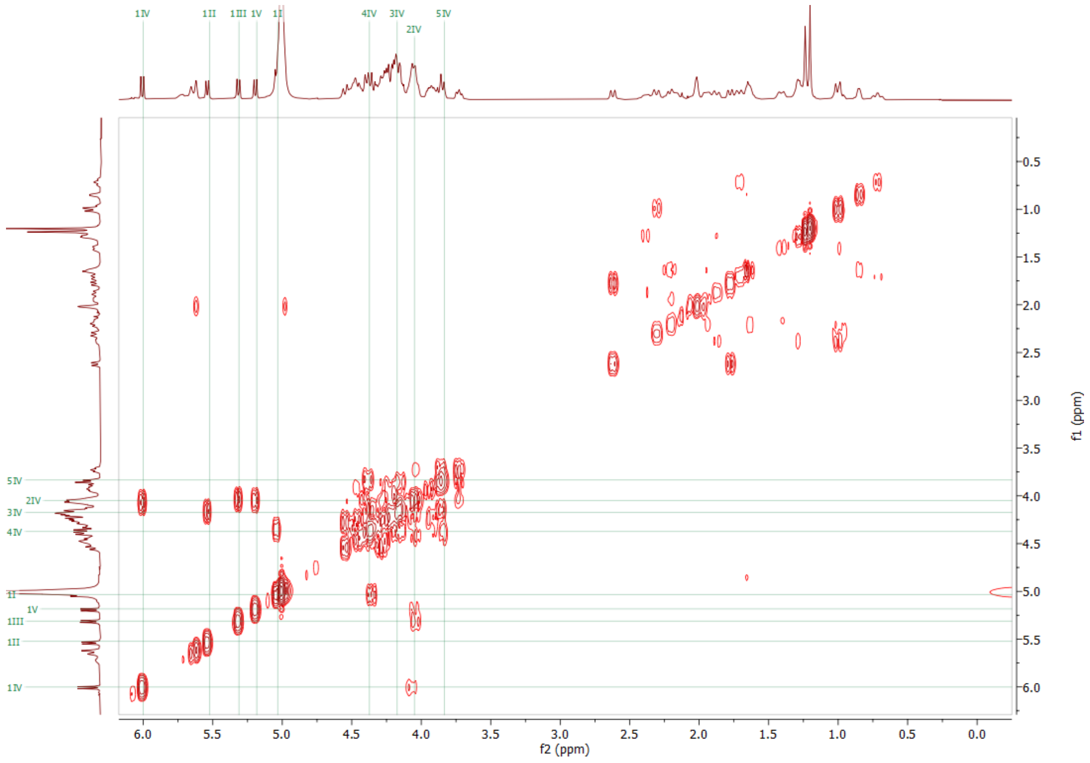

Supplementary Figure S8

Figure S8. ¹H–¹³C HSQC-DEPT NMR spectrum of Rebaudioside *D17* (Sugar region and aglycone region).

The spectrum includes both sugar and aglycone regions of Rebaudioside *D17*.

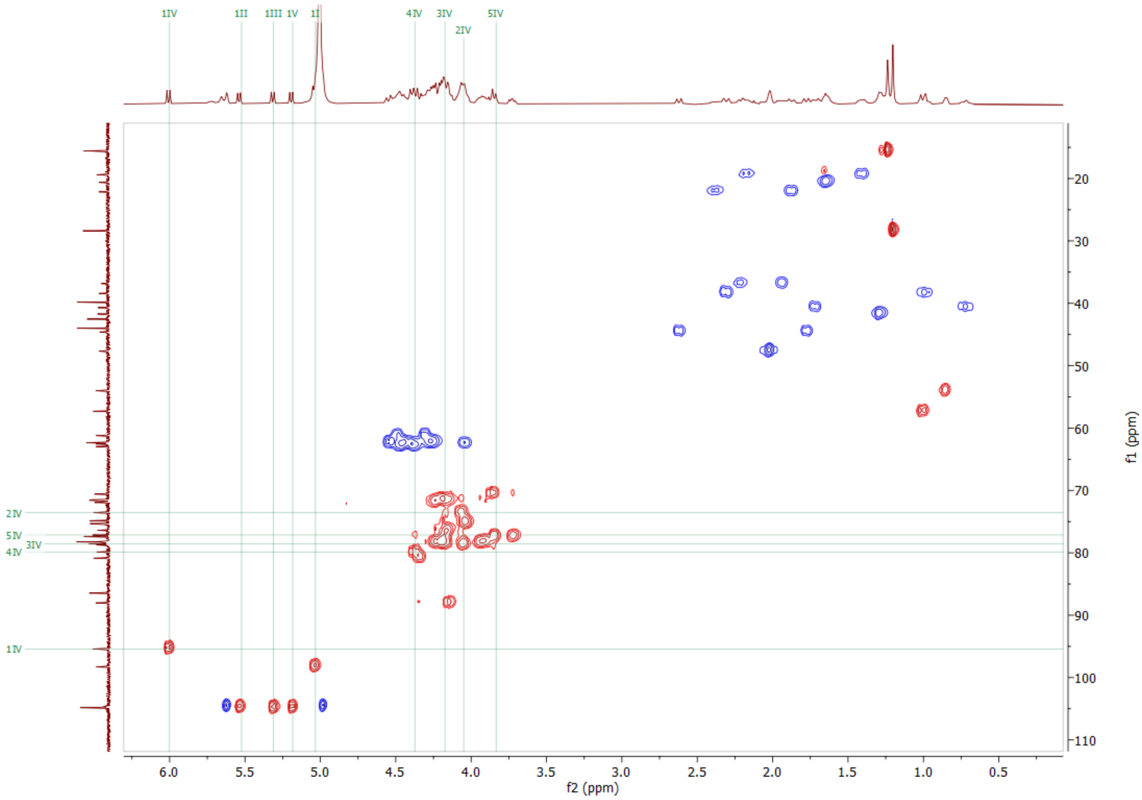

Supplementary Figure S9

Figure S9. ^1H–^{13}C HMBC NMR spectrum of Rebaudioside *D17* (Sugar region and aglycone regions).

The spectrum includes both sugar and aglycone regions of Rebaudioside *D17*. Key HMBC correlations in the sugar region supporting the structural assignment of the β-1,4 glycosidic linkage are annotated and discussed in the Results section.

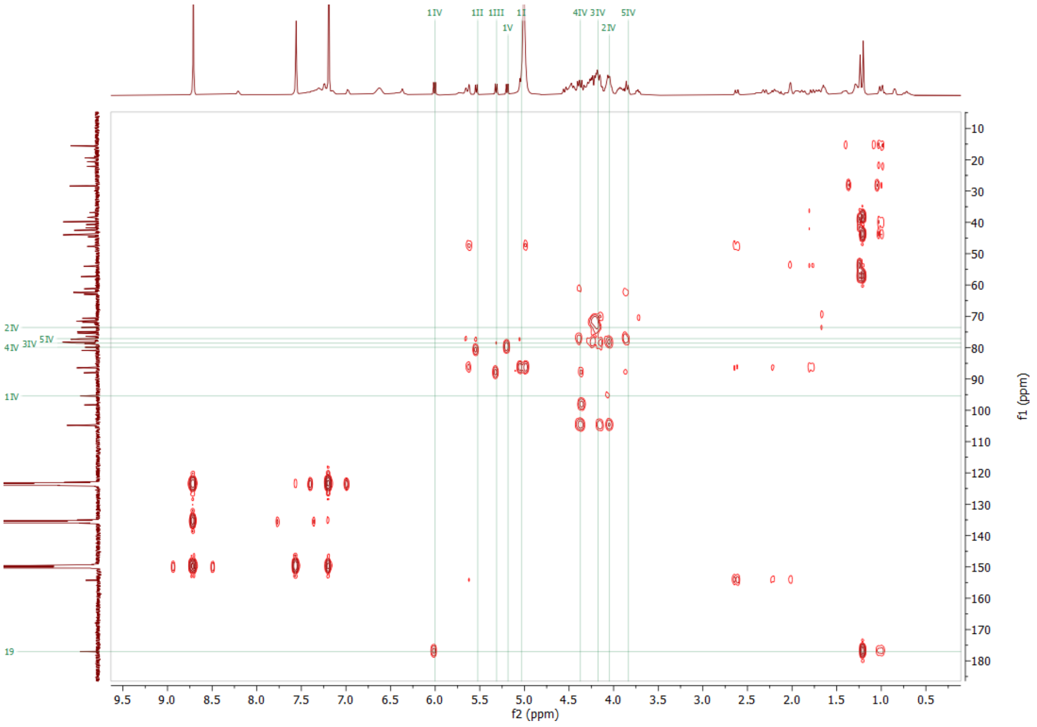

Supplementary Figure S10

Figure S10. ^1H–^{13}C HSQC-TOCSY NMR spectrum of Rebaudioside *D17* (Sugar region).

The spectrum focuses on the sugar region of Rebaudioside *D17*. Key cross-peaks for sugar IV are annotated, supporting the structural assignment of the β-1,4 glycosidic linkage.

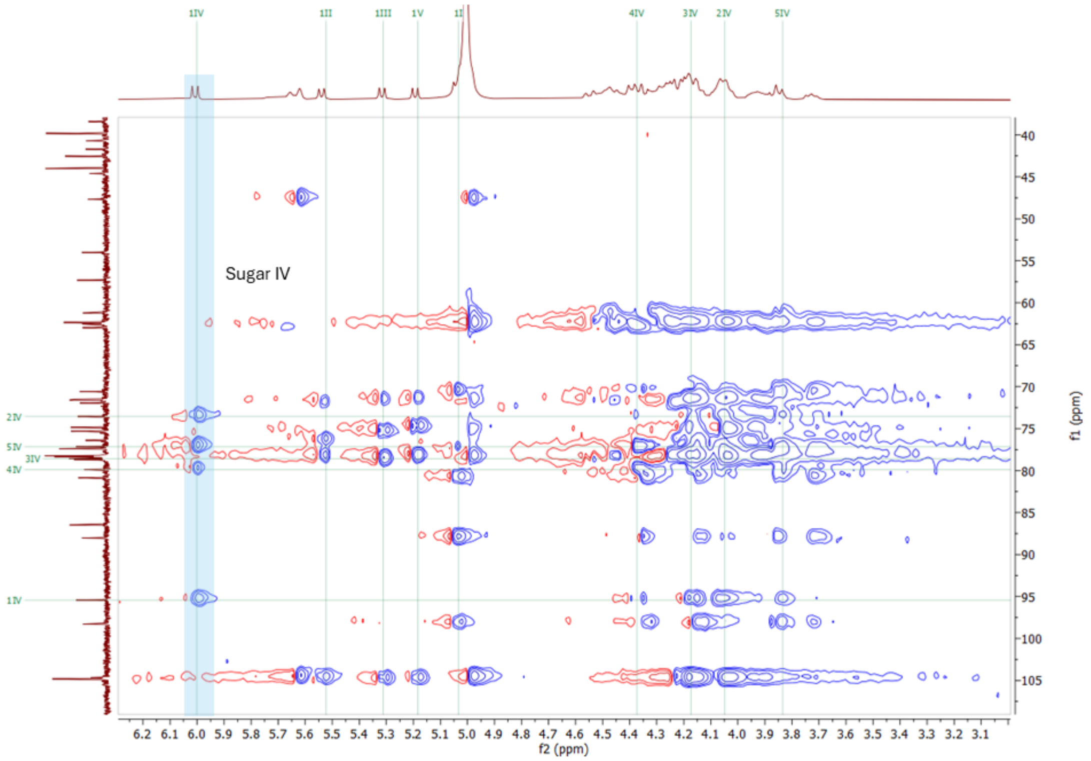

Supplementary Figure S11

Figure S11. ^1H–^1H NOESY NMR spectrum of Rebaudioside *D17*.

Correlations between H-1^{IV} and H-3^{IV}, as well as H-1^{IV} and H-5^{IV}, are annotated. These NOE interactions confirm the identity of Sugar IV as β-D-glucose. Similar analysis was performed for the remaining sugar units, all of which were likewise confirmed to be β-D-glucoses.

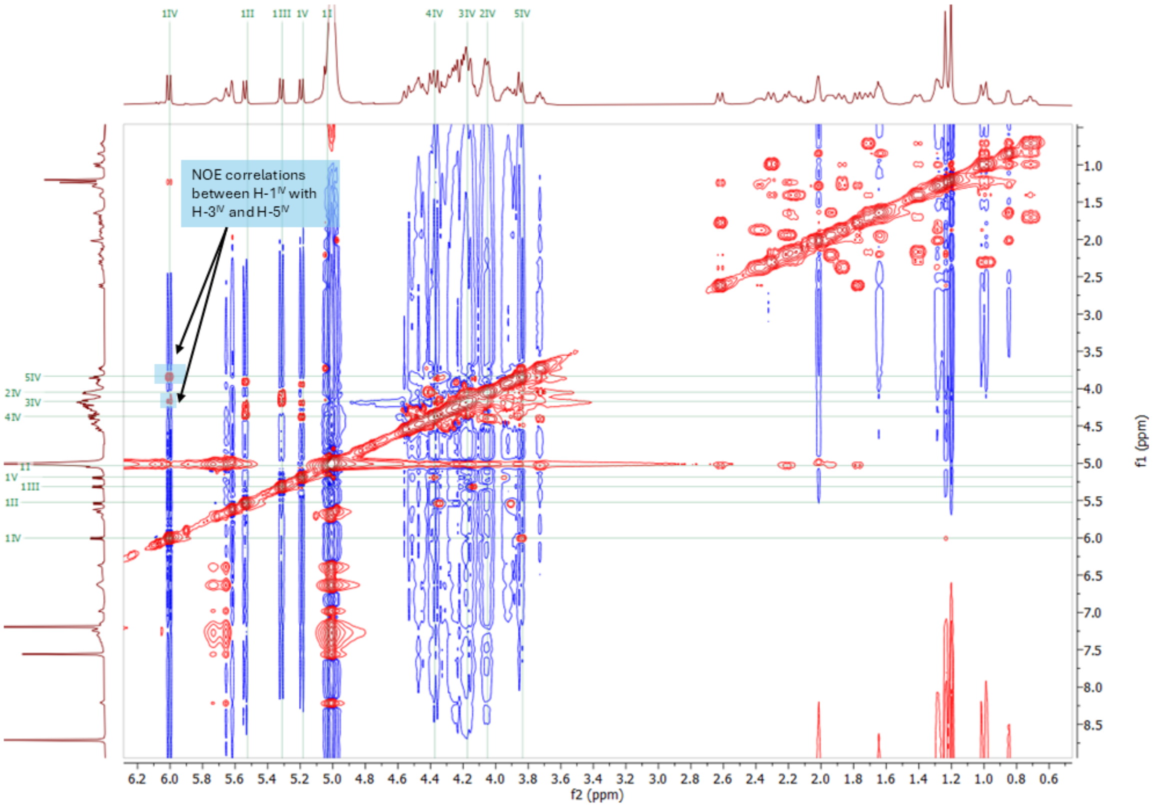

Supplementary Table S1

Table S1. ^{1}H and ^{13}C NMR chemical shift data for the aglycone of Rebaudioside *D17*.

^{1}H and ^{13}C NMR chemical shift data (400 MHz, pyridine-d_5) for the aglycone of Rebaudioside *D17*, including chemical shifts (δ), coupling constants (*J*) and integration values.

Position	δ_C [ppm]	δ_H [ppm]	Integration	*J* [Hz]
1	40.3	0.72 m, 1.72 m		
2	19.1	1.41 m, 2.17 m		
3	38.1	0.99 m, 2.31 m		
4	43.9	-		
5	57.1	1.01 m		
6	21.8	1.88 m, 2.38 m		
7	41.4	1.30 m	2H	
8	40.4	-		
9	53.7	0.85 m		
10	41.4	-		
11	20.3	1.65 m	2H	
12	36.6	1.94 m, 2.21 m		
13	86.3	-		
14	44.2	1.78 d, 2.62 d		11.0, 11.0
15	47.4	2.02 m	2H	
16	153.9	-		
17	104.4	4.89 br s, 5.62 br s		
18	28.2	1.20 s	3H	
19	176.8	-		
20	15.3	1.24 s	3H	

Supplementary Table S2

Table S2. ^{1}H and ^{13}C NMR chemical shift data for the sugar moieties of Rebaudioside *D17*.

^{1}H and ^{13}C NMR chemical shift data (400 MHz, pyridine-d_5) for the sugar moieties of Rebaudioside *D17*, including chemical shifts (δ), coupling constants (*J*) and key ^{1}H–^{1}H NOESY and ^{1}H–^{13}C HMBC correlations.

Position	δ_C [ppm]	δ_H [ppm]	*J* [Hz]	HMBC (H→C)	NOESY (H → H)
		**Sugar I: β-D-Glucopyranoside**			
1^{I}	98.0	5.03 d	8.0	13	3^{I}, 5^{I}
2^{I}	80.7	4.35 m			
3^{I}	87.9	4.14 m			
4^{I}	70.3	3.86 m			
5^{I}	77.2	3.71 m			
6^{I}	62.2	4.04 m, 4.41 m			
		**Sugar II: β-D-Glucopyranoside**			
1^{II}	104.6	5.53 d	8.0	2^{I}	3^{II}, 5^{II}
2^{II}	76.1	4.16 m			
3^{II}	78.1	4.23 m			
4^{II}	71.7	4.25 m			
5^{II}	77.9	3.90 m			
6^{II}	62.5	4.38 m, 4.47 m			
		**Sugar III: β-D-Glucopyranoside**			
1^{III}	104.6	5.32 d	8.0	3^{I}	3^{III}, 5^{III}
2^{III}	74.9	4.04 m			
3^{III}	78.1	4.19 m			
4^{III}	71.3	4.20 m			
5^{III}	78.4	4.06 m			
6^{III}	62.1	4.27 m, 4.47 m			
		**Sugar IV: β-D-Glucopyranoside**			
1^{IV}	95.2	6.01 d	8.0	19	3^{IV}, 5^{IV}
2^{IV}	73.4	4.07 m			
3^{IV}	78.2	4.18 m			
4^{IV}	79.6	4.38 m			
5^{IV}	77.1	3.85 m			
6^{IV}	60.9	4.31 m, 4.50 m			
		**Sugar V: β-D-Glucopyranoside**			
1^{V}	104.6	5.20 d	8.0	4^{IV}	3^{V}, 5^{V}
2^{V}	74.9	4.06 m			
3^{V}	77.9	4.19 m			
4^{V}	71.3	4.16 m			
5^{V}	78.0	3.94 m			
6^{V}	62.1	4.42 m, 4.53 m			
